# TRIM22 confers poor prognosis and promotes epithelial-mesenchymal transition through regulation of AKT/GSK3β/β-catenin signaling in non-small cell lung cancer

**DOI:** 10.18632/oncotarget.18911

**Published:** 2017-07-01

**Authors:** Li Liu, Xiao-Ming Zhou, Fang-Fei Yang, Yuan Miao, Yan Yin, Xue-Jun Hu, Gang Hou, Qiu-Yue Wang, Jian Kang

**Affiliations:** ^1^ Department of Respiratory Medicine, The First Hospital of China Medical University, Shenyang 110001, China; ^2^ Department of Respiratory Medicine, The Shengjing Hospital of China Medical University, Shenyang 110004, China; ^3^ Department of Pathology, The First Hospital and College of Basic Medical Sciences, China Medical University, Shenyang 110001, China

**Keywords:** TRIM22, prognosis, non-small cell lung cancer, proliferation, invasion

## Abstract

Expression pattern and biological roles of TRIM22 remains unknown in most human cancers. The present study aims to discover its clinical significance and function in human non-small cell lung cancer (NSCLC). Immunohistochemistry was used to examine TRIM22 expression in 126 cases of NSCLC specimens. TRIM22 protein was upregulated in 70/126 (55.6%) non-small cell lung cancer tissues compared with normal lung tissue. TRIM22 overexpression was associated with advanced TNM stage, positive nodal metastasis and poor prognosis. Plasmid and siRNA transfection were performed in lung cancer cell lines. TRIM22 overexpression promoted proliferation, colony formation and invasion in A549 cells. While its depletion exhibited the opposite effects in H1299 cell line. TRIM22 overexpression promoted cell cycle progression through regulation of cyclin D1, cyclin E and p27. TRIM22 also changed the expression of epithelial to mesenchymal transition (EMT) markers including E-cadherin N-cadherin, Vimentin and Snail. Furthermore, TRIM22 activated PI3K/AKT/GSK3β/β-catenin oncogenic signaling pathways. Treatment with PI3K inhibitor LY294002 and β-catenin siRNA blocked the effects of TRIM22 on EMT in TRIM22-overexpressing cells. In conclusion,TRIM22 serves as an important oncoprotein and a promoter of cell proliferation and invasion through AKT/ GSK3β/β-catenin induced EMT in NSCLC.

## INTRODUCTION

Lung cancer is the leading cause of cancer-related death worldwide and its incidence is increasing [1–3]. Non-small cell lung cancer (NSCLC) is the main histological type, accounting for 85% of lung cancer cases. Despite recent advances in diagnosis and treatment including surgery, chemotherapy, radiotherapy and targeted therapy, the prognosis of NSCLC patient remains poor [4–10]. It is necessary to identify new molecular markers involved in the regulation of lung cancer cell aggressiveness.

TRIM22 is a member of the TRIM family proteins which contain RING finger, B-box, and coiled-coil domains. It has been reported as a transcriptional regulator which was revolved in many biological processes. TRIM22 was also reported to functions as a E3 ubiquitin ligase [11]. A study identified TRIM22 as a progesterone responsive gene in Ishikawa endometrial cancer cells [12]. The role of TRIM22 in human cancer was seldom reported. It has been shown that TRIM22 was downregulated in breast cancer and associated with a lack of p53 mediated induction [13]. A recent report identified TRIM22 mRNA was upregulated in NCSLC cell lines using Q-PCR [14], suggesting its potential involvement in cancer development. Thus, the role of TRIM22 seems controversial. In addition, its protein expression and potential biological roles remains unclear in most human cancers.

In the present study, we examined clinical significance of TRIM22 protein in 126 cases of NSCLCs. We also examined its biological function in NSCLC cell lines. We further investigated the underlying mechanisms of its biological effects in NSCLC cells.

## RESULTS

### TRIM22 is overexpressed in NSCLC

**Figure 1:**
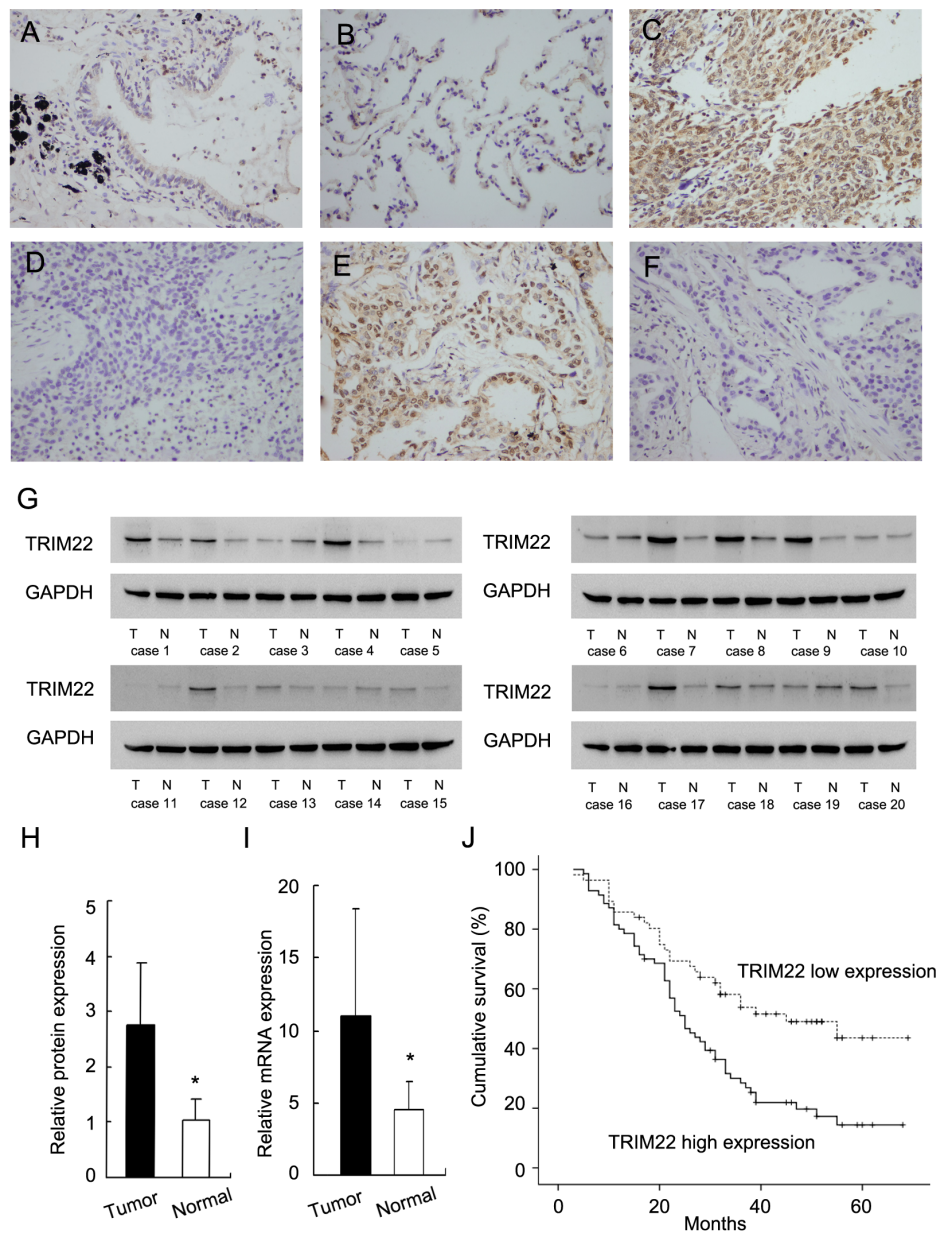
Expression of TRIM22 in non-small cell lung cancers **(A)** Negative staining of TRIM22 in normal bronchial epithelial cells. **(B)** Negative staining of TRIM22 in normal alveolar cells. **(C)** Strong nuclear staining of TRIM22 in lung squamous cell carcinoma. **(D)** Negative TRIM22 expression in squamous cell carcinoma. **(E)** Strong nuclear staining of TRIM22 in lung adenocarcinoma. **(F)** Negative TRIM22 expression in lung adenocarcinoma. **(G)** Western blot analysis showed TRIM22 protein was significantly elevated in cancer tissues compared with corresponding normal tissues (50 kda). **(H)** Protein quantification of TRIM22 expression in cancer/normal tissues using ImageJ software. **(I)** Real-time PCR showed that mean TRIM22 mRNA level in lung cancer tissues was higher than that in paired adjacent normal tissues. **(J)** Survival analysis of patients with different TRIM22 status. The overall survival was significantly lower in patients with high TRIM22 expression than in patients with low TRIM22 expression. * P<0.05.

TRIM22 protein was examined in 126 cases of NSCLC cancer specimens and 15 cases of normal lung tissue specimens using immunohistochemistry.In normal lung tissues, we observed weak/negative TRIM22 staining in normal bronchial epithelial cells and alveolar cells (Figure 1A–1B). TRIM22 staining can be observed in both nuclear and cytoplasmic compartment of tumor cells. Immunocytochemistry using H1299 cells also showed nuclear and cytoplasmic staining of TRIM22 (Supplement Figure 1A). Since TRIM22 was reported as a nuclear protein, we considered nuclear TRIM22 staining as positive. As for lung cancer tissues, 55.6% (70/126) cases showed high TRIM22 expression (Figure 1C–1F) while the rest showed weak/negative staining. We analyzed the correlation between TRIM22 status and clinicopathological factors. As shown in Table 1, we found that TRIM22 overexpression significantly correlated with advanced TNM stage (stage ш vs stage П vs stage І: 42% vs 54.3% vs 73.2%, p=0.0117) and positive nodal status (p=0.0076). No difference was found between TRIM22 status with other parameters including age, gender, histology, differentiation and T stage (Table 1). Cytoplasmic TRIM22 staining was also analyzed. Cytoplasmic TRIM22 overexpression also correlated with advanced stage and nodal metastasis (Supplement Table 1). We validated TRIM22 status in fresh lung cancer tissues. Western blot analysis showed significant TRIM22 protein overexpression in lung cancer tissues (Figure 1G). Relative protein expression was quantified using ImageJ software. Expression of TRIM22 in cancer tissues is 2.58 fold higher than that in normal tissues (Figure 1H). Real-time PCR analysis showed that TRIM22 mRNA level was 2.42 fold higher in cancer tissues compared with that in adjacent normal tissues (Figure 1I).

**Table 1:** Expression of TRIM22 in NSCLC.

Characteristics	Number of patients	TRIM22 low expression	TRIM22 high expression	*P*
Age				
<60	54	25	29	0.7171
≥60	72	31	41	
Gender				
Male	85	39	46	0.6400
Female	41	17	24	
Differentiation				
Poor	16	9	7	0.5498
Moderate	84	35	49	
Well	26	12	14	
Histology				
Adenocarcinoma	70	30	26	0.0652
Squamous cell carcinoma	56	26	44	
TNM stage				
I	50	29	21	0.0117
II	35	16	19	
III	41	11	30	
T stage				
T1	31	13	18	0.7461
T2-T4	95	43	52	
Nodal metastasis				
Negative	62	35	27	0.0076
Positive	64	21	43	

In addition, we analyzed the correlation of TRIM22 with patient prognosis. Kaplan-Meier survival analysis demonstrated that patients with high TRIM22 expression had significantly lower overall survival compared with those with low TRIM22 expression (p=0.0011, Log-Rank test; Figure 1J). As shown in Table 2 , univariate and multivariate Cox model indicated that both TNM stage and TRIM22 status were independent, unfavorable prognostic factors (Univariate analysis: TNM stage, p<0.001; TRIM22, p=0.0016; multivariate analysis: TNM stage, p<0.001; TRIM22, p=0.0329).

**Table 2:** Univariate and multivariate analysis for predictive factors in patients with NSCLC

	Univariate		Multivariate	
Factors	Hazard ratio (95% CI)	p Value	Hazard ratio (95% CI)	p Value
Gender	1.088 (0.681-1.737)	0.7235	1.051 (0.644-1.715)	0.8418
Age	0.808 (0.525-1.245)	0.3338	0.708 (0.442-1.135)	0.1514
Histology	0.955 (0.621-1.470)	0.8351	0.883 (0.569-1.371)	0.5804
Differentiation	0.830 (0.578-1.191)	0.3122	0.752 (0.497-1.138)	0.1777
TNM Stage	2.086 (1.589-2.737)	<0.001	1.822 (1.359-2.442)	<0.001
TRIM22	2.081 (1.319-3.285)	0.0016	1.734 (1.046-2.874)	0.0329

### TRIM22 overexpression promotes proliferation and colony formation of lung cancer cells

**Figure 2:**
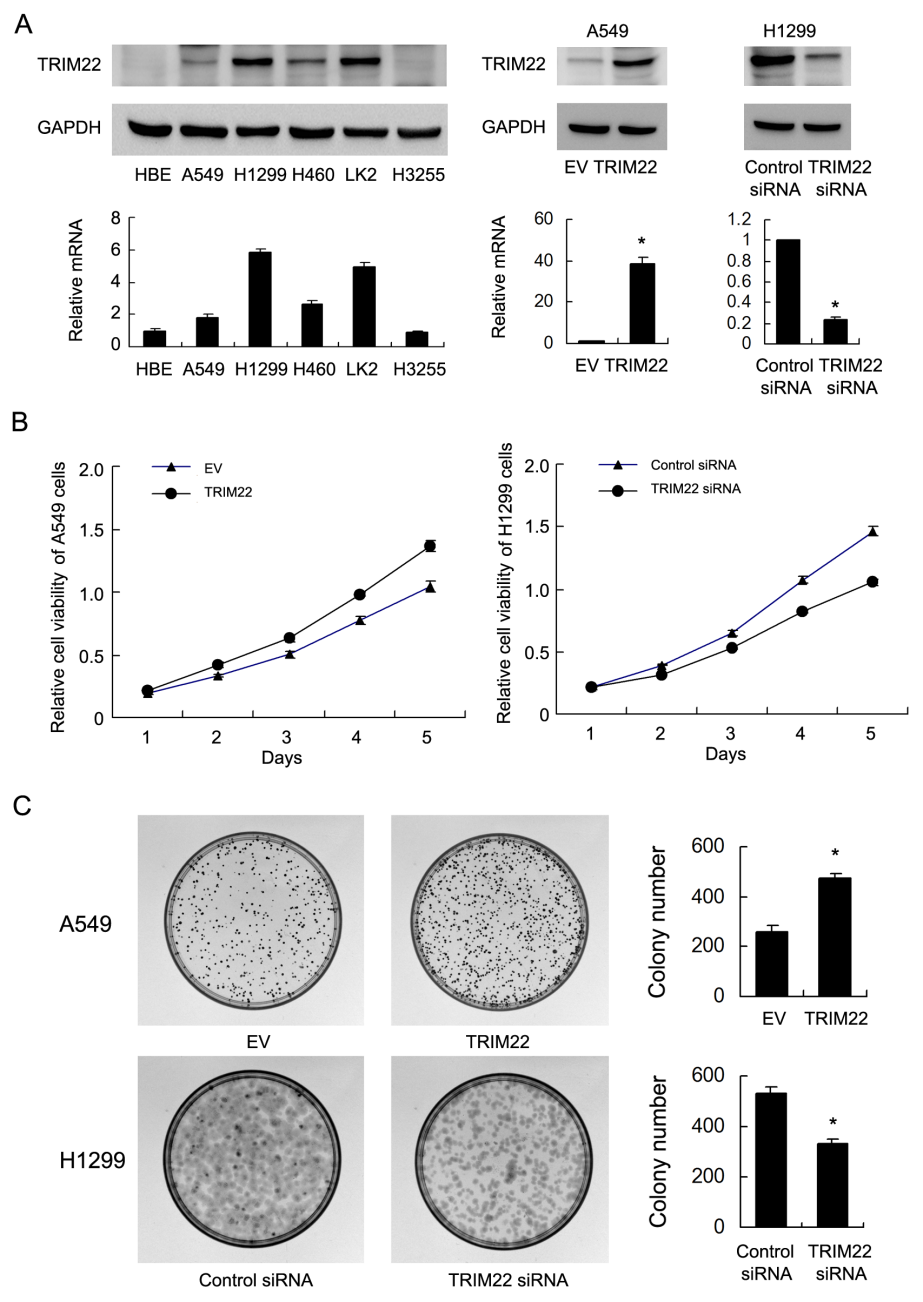
TRIM22 expression in lung cancer cell lines and its role on proliferation and colony formation **(A)** Endogenous expression of TRIM22 was examined in HBE and lung cancer cell lines by western blot and RT-qPCR. Lung cancer cell lines have significant upregulated TRIM22 expression. Western blot analysis showed that pCMV6-TRIM22 plasmid markedly increases its levels in A549 cells compared with control. TRIM22 siRNA downregulated its expression in H1299 cells. (B) MTT showed that TRIM22 overexpression in A549 cells greatly promoted the proliferation rate while TRIM22 depletion inhibited proliferation rate. (C) TRIM22 overexpression in A549 cells promoted the colony formation ability while TRIM22 depletion inhibited colony formation ability. * P<0.05.

Relative expression level of TRIM22 was analyzed in a panel of lung cancer cell lines. Western blot and realtime RT-PCR showed that TRIM22 protein expression was remarkably increased in NSCLC cell lines compared with normal HBE cell line, especially H1299 and LK2 cell lines (Figure 2A). We selected A549 cell line with low endogenous expression for TRIM22 plasmid transfection. H1299 cell line was used for siRNA treatment. Transfection efficiency was confirmed by western blot and PCR analysis (Figure 2A). Then we examined the change of proliferation rate by MTT assay and colony formation assay (Figure 2B–2C). TRIM22 overexpression in A549 cells greatly promoted the proliferation rate and the potential of colony formation (A549 EV vs. TRIM22: 260±23 vs. 471±22, p<0.05), while TRIM22 depletion in H1299 cells inhibited proliferation and colony formation ability (H1299 Neg siRNA vs. TRIM22 siRNA: 534±21 vs. 331±20, p<0.05).

### TRIM22 regulates cell cycle and related proteins

**Figure 3:**
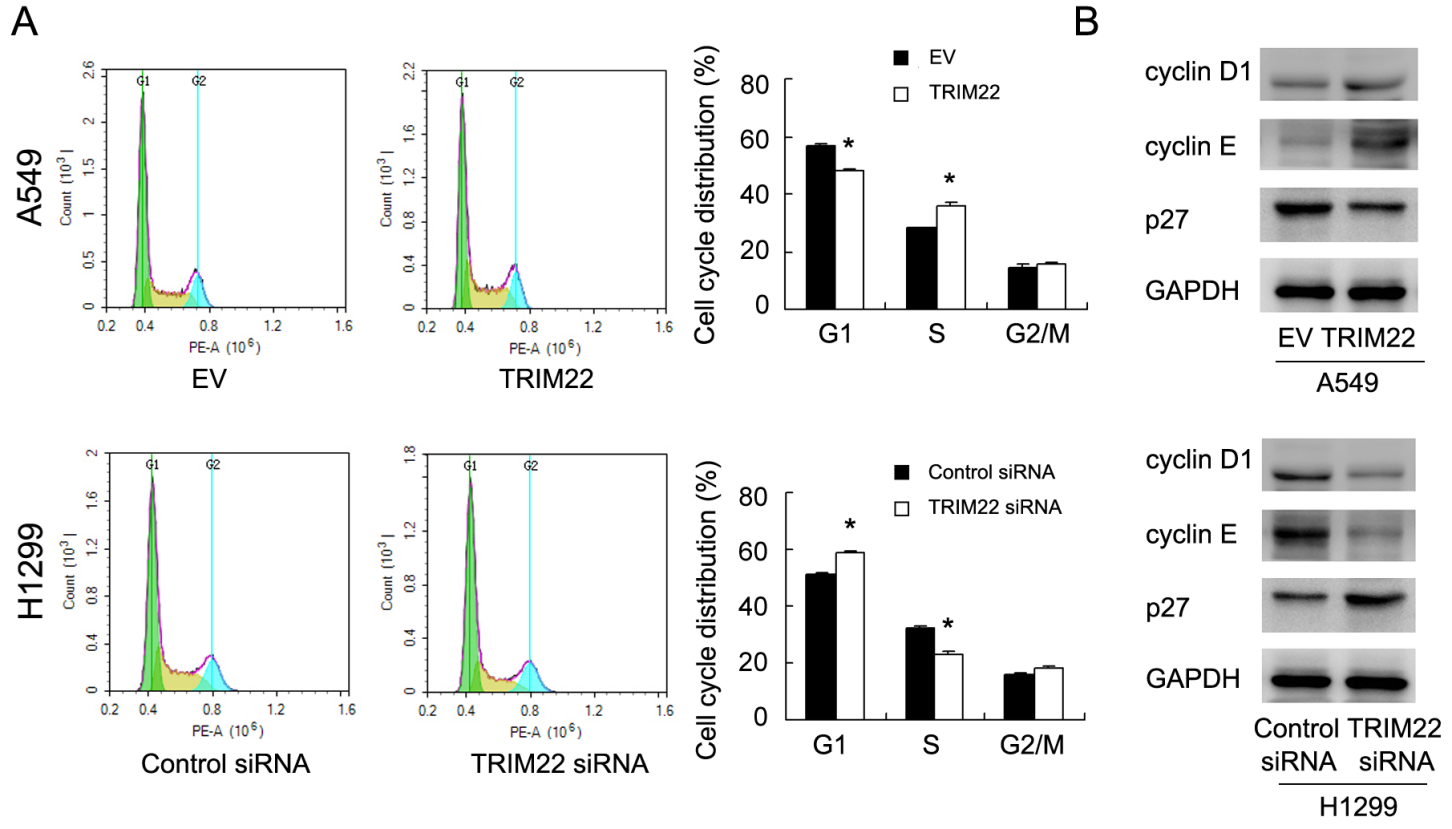
TRIM22 promotes cell cycle progression **(A)** Cell cycle analysis showed that TRIM22 transfection increased percentage of S phase and decreased the percentage of G1 phase. TRIM22 depletion exhibited the opposite effects. **(B)** Western blot analysis showed that TRIM22 overexpression increased the protein expression of cyclin D1 (36 kda), cyclin E (48 kda), and decreased p27 (27 kda) expression in A549 cells. TRIM22 depletion showed the opposite effects in H1299 cells. * P<0.05.

Since TRIM22 could regulate cancer cell proliferation, we checked the change of cell cycle progression. Figure 3A showed that TRIM22 overexpression facilitated G1-S progression in A549 cell line, with upregulation of S phase percentage and downregulation of G1 phase percentage. TRIM22 siRNA inhibited cell cycle transition in H1299 cell line, downregulating S phase percentage and upregulating G1 phase percentage. We also checked several cell cycle related proteins. TRIM22 transfection significantly increased expression cyclin D1, cyclin E, and reduced cell cycle inhibitor p27 expression. TRIM22 depletion in H1299 cells demonstrated the opposite effects on these proteins (Figure 3B).

### TRIM22 regulates cell invasion and E-cadherin expression

**Figure 4:**
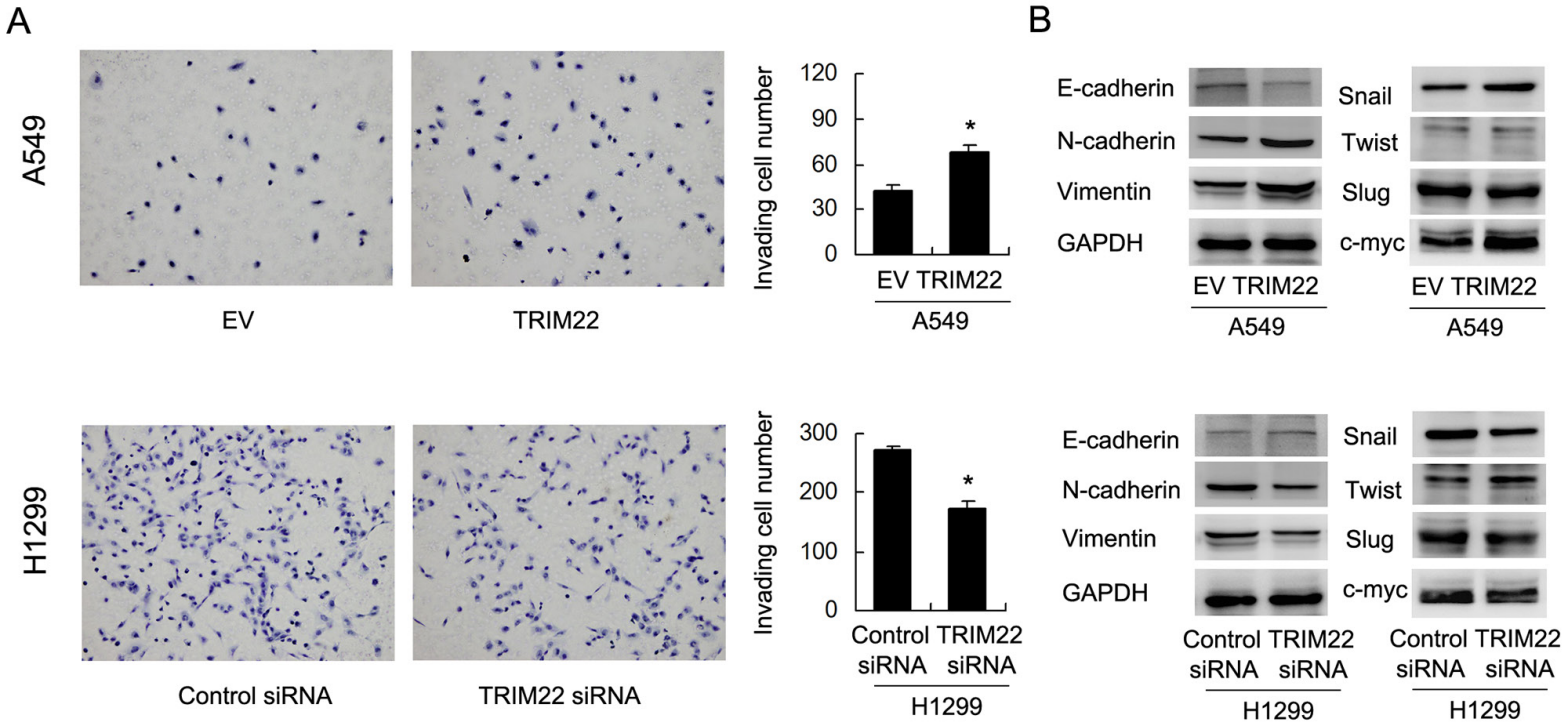
TRIM22 promotes cell invasion and EMT **(A)** TRIM22 overexpression in A549 cells greatly promoted cell invasion while TRIM22 depletion inhibited invading ability of H1299 cells. **(B)** Western blot results showed that TRIM22 transfection downregulated E-cadherin (135 kda) and upregulated N-cadherin (140 kda), Vimentin (57 kda), Snail (29kda), c-myc (57 kda) in A549 cells. TRIM22 depletion in H1299 cell line upregulated E-cadherin and downregulated N-cadherin, Vimentin, Snail expression. The change of Slug (30 kda) and Twist (21 kda) was not obvious. * P<0.05.

To clarify the biological effect of TRIM22 on cell invasion, we carried out Matrigel invasion assay. As shown in Figure 4A, TRIM22 transfection significantly increased cell invasion in A549 cells (A549 EV vs. TRIM22: 42.3±3.5 vs. 68.1±4.3, p<0.05). TRIM22 depletion in H1299 cells reduced invading ability (H1299 Neg siRNA vs. TRIM22 siRNA: 271.6±6.5 vs. 172.6±13.6, p<0.05). TRIM22 also positively regulated cell migration rate in lung cancer cells (Supplement Figure 1B). To further explore the potential mechanism of TRIM22 in the regulation of lung cancer invasion, we checked epithelial-mesenchymal transition (EMT) related proteins. Western blot results showed that TRIM22 transfection downregulated E-cadherin and upregulated N-cadherin, Vimentin in A549 cells. TRIM22 depletion in H1299 cell line upregulated E-cadherin and downregulated N-cadherin, Vimentin expression (Figure 4B). In addition, we found that TRIM22 could induce Snail and c-myc expression while its depletion inhibited its level.

### TRIM22 regulates epithelial-mesenchymal transition through activation of PI3K/AKT signaling

**Figure 5:**
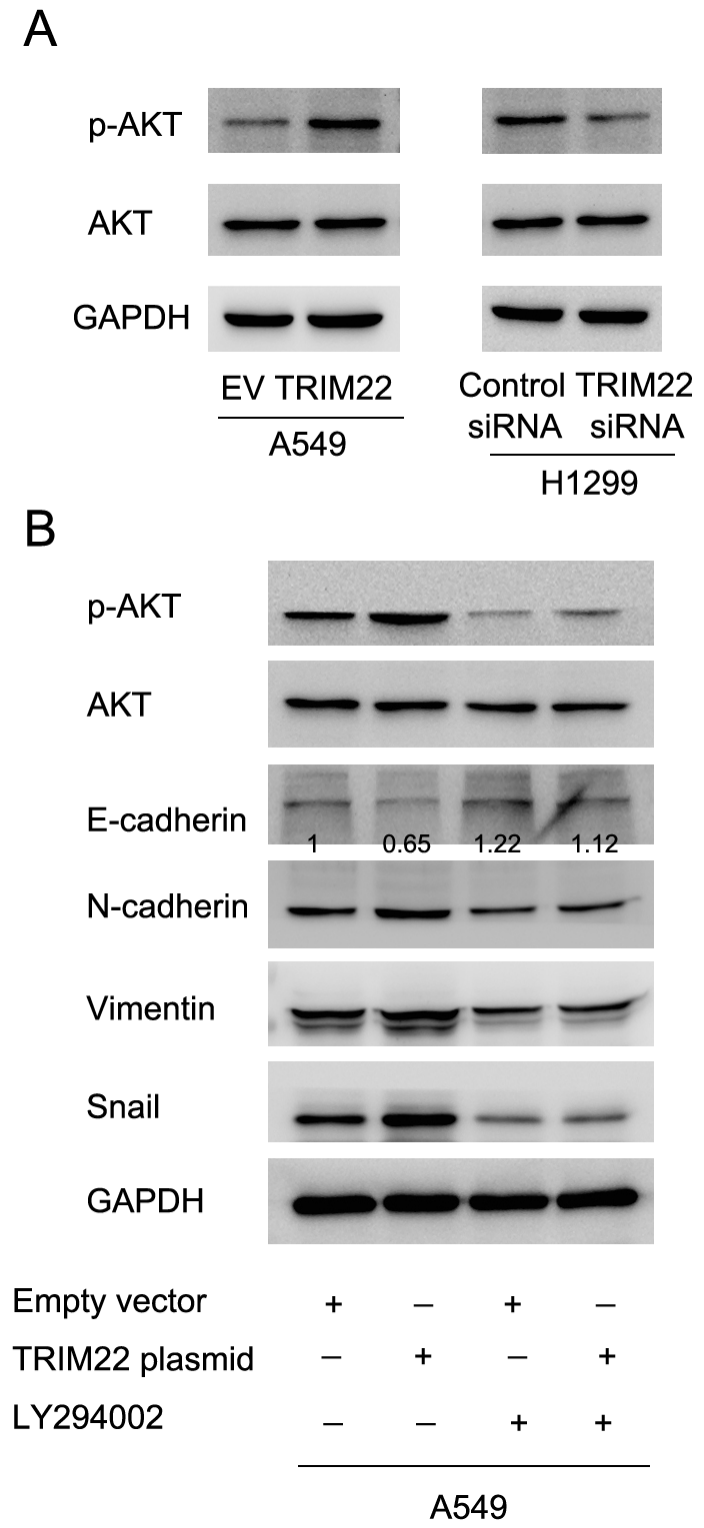
TRIM22 regulates epithelial-mesenchymal transition through activation of PI3K/AKT signaling **(A)** TRIM22 overexpression in A549 cell line resulted in upregulation of phsopho-AKT (70 kda). TRIM22 depletion decreased p-AKT level. **(B)** A549 cells was treated with LY294002 (10μM, 24h), which blocked expression of p-AKT. In cells treated with LY294002, TRIM22 overexpression did not cause significant change of E-cadherin, N-cadherin, Vimentin and Snail. * P<0.05.

Next we sought the underlying mechanism of TRIM22 induced EMT. After screening a series of related signaling pathways, we found that TRIM22 transfection upregulated expression of phospho-AKT while its depletion downregulated AKT phsophorylation (Figure 5A). Since AKT activation has been reported in the development of EMT [15], we sought to validate their interaction by using LY294002, an inhibitor of PI3K/AKT signaling. We treated A549 cells with LY294002 (10μM, 24h) and then test the change of related molecules. As shown in Figure 5B, AKT inhibitor significantly blocked AKT phsophorylation. AKT inhibitor also abolished the effect of TRIM22 on EMT markers. In A549 cells treated with LY294002, TRIM22 overexpression failed to change the expression of E-cadherin, N-cadherin, Vimentin and Snail, suggesting AKT activation is responsible for the role of TRIM22 on EMT.

### TRIM22 activates AKT/ GSK3β/β-catenin signaling to induce EMT in lung cancer cells

**Figure 6:**
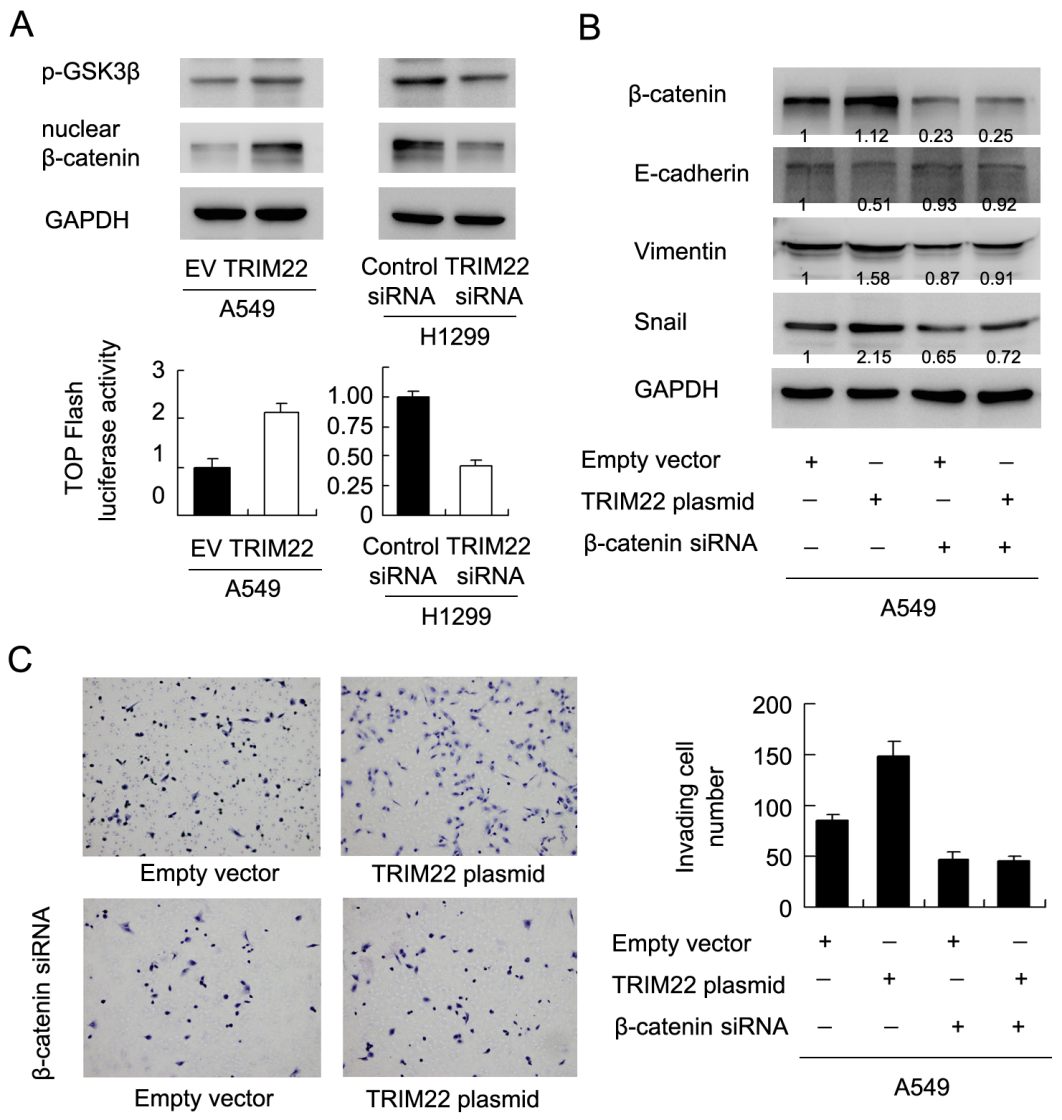
TRIM22 activates AKT/GSK3β/β-catenin signaling to induce EMT **(A)** TRIM22 upregulated GSK3β(46 kda) phosphorylation and nuclear β-catenin (92 kda)protein in A549 cells. TRIM22 depletion downregulated GSK3β phosphorylation and nuclear β-catenin protein in H1299 cells. TOP-Flash assay showed that TRIM22 overexpression upregulated Wnt activity while its depletion downregulated Wnt activity. **(B)** β-catenin siRNA depleted its endogenous expression in A549 cells. In A549 cells with β-catenin siRNA, E-cadherin downregulation and vimentin/Snail upregulation did not occur after TRIM22 treatment. **(C)** TRIM22 promoted A549 cell invasion. While in β-catenin depleted cells, TRIM22 failed to upregulate invading ability.

AKT activation could elevate GSK3β phosp-horylation, which in turn induces Wnt signaling activity and promotes EMT in human cancers. Using western blot and luciferase reporter (Figure 6A), we found that TRIM22 could activate GSK3β phosphorylation, which lead to accumulation of nuclear β-catenin protein and upregulation of Wnt activity measured by TOP-Flash assay. To validate the involvement of Wnt signaling in the EMT process induced by TRIM22, we adopted β-catenin siRNA to block Wnt activation in A549 cells. As shown in Figure 6B, β-catenin siRNA showed significant knockdown effect on its protein expression. In cells with β-catenin siRNA, E-cadherin downregulation and vimentin/Snail upregulation did not occur after TRIM22 treatment. In addition, β-catenin depleted cells did not display significant change in the invading ability after TRIM22 overexpression (Figure 6C). We also checked the effect of β-catenin siRNA on A549 proliferation. As shown in Supplement Figure 1C, β-catenin siRNA downregulated the growth promoting effect of TRIM22.

## DISCUSSION

There have been two studies concerning TRIM22 in human cancers. One reported showed TRIM22 downregulationg in breast cancer cell lines and tissues compared with non-malignant mammary epithelial cell lines and normal breast tissues [13]. A recent study compared TRIM family mRNAs in non-small cell lung cancer and normal bronchial epithelial cell line HBE. TRIM22 was one of the 10 TRIM genes upregulated in NSCLC cell lines [14]. Thus the function and status of TRIM22 in human cancer is quite vague and might be tissue specific. To validate TRIM22 status in NSCLC, immunohistochemistry was carried out to examine its protein status in 126 cases of NSCLC tissues. For the first time, we showed that TRIM22 protein expression was significantly upregulated in 55.6% lung cancer specimens examined, which significantly correlated with positive lymph node metastasis and advanced TNM stage. Importantly, we showed that TRIM22 overexpression correlated poor survival of lung cancer patients. TRIM22 could also serve as an independent predicting factor for patient prognosis. These data suggest TRIM22 could serve as a potential oncoprotein in NSCLC and an predicting factor for poor patient survival.

The western blot and RT-qPCR data also demonstrated TRIM22 overexpression in NSCLC cell lines at both mRNA and protein levels, which is consistent with previous report [16]. We further adopted TRIM22 plasmid and siRNA to examine its biological function. MTT, colony formation assay demonstrated that TRIM22 overexpression promoted *in vitro* cell growth. Cell cycle analysis showed that TRIM22 could facilitate G1-S cell cycle transition. Depletion of endogenous TRIM22 exhibited the opposite effects. These data validate TRIM22 as growth promoter through regulation of cell cycle progression in NSCLC cell lines. We also found cell cycle related proteins cyclin D1 and cyclin E were significantly upregulated by TRIM22. The expression of p27 was downregulated after TRIM22 overexpression. Cyclin D1 and cyclin E are important members of cyclin family, which are upregulated in human lung cancer cells and regulates the progression of the cell cycle by controlling G1/S transition [17–20]. p27 is frequently downregulated in tumor cells, which functions as a CDK inhibitor and causes cell cycle arrest in the G1 phase [21–24]. These results was in accordance with the fact that TRIM22 could facilitate cell cycle transition, indicating a oncogenic function of TRIM22 in lung cancer cells.

Epithelial to mesenchymal transition (EMT) is a vital process in the conversion of early-stage tumors into invasive malignancies. It was shown that the EMT is associated with lung cancer invasion and metastasis [25, 26]. EMT process is characterized by upregulation the mesenchymal markers such as N-cadherin and downregulation of epithelial marker like E-cadherin, leading to the disruption of cell junctions [27]. In this study, we demonstrated that TRIM22 promoted invading ability of lung cancer cells using matrigel invasion assay. When exploring its underlying mechanism, we found that TRIM22 downregulated E-cadherin and upregulated N-cadherin. In contrast, TRIM22 siRNA reversed this process, suggesting that TRIM22 could be a novel promoter of EMT process in lung cancer cells. The transcription factor Snail controls epithelial-mesenchymal transitions by repressing E-cadherin expression. Our results showed that TRIM22 could induce Snail expression in lung cancer cells, suggesting TRIM22 control EMT process through induction of Snail.

PI3K/AKT signaling is involved in a broad variety of biological functions, including proliferation, differentiation, survival, and motility. Several studies indicated that the engagement of EMT process in activation of AKT in epithelial cells and carcinoma cells [28, 29]. In the present study, we found that the expression of p-AKT was significantly upregulated after TRIM22 overexpression. We also treated A549 cells with the inhibitor of PI3K/AKT signaling. Several studies has demonstrated the association of Snail and PI3K/AKT in human cancers [30–33]. We postulated that TRIM22 suppressed E-cadherin by AKT mediated Snail induction. To validate this, we adopted a potent AKT inhibitor to block the activation of AKT and then test change of EMT markers. The results showed that AKT inhibitor blocked the effects of TRIM22 on Snail and EMT markers.

It has been reported that AKT activates GSK3β phosphorylation, which leads to β-catenin nuclear accumulation [34]. Nuclear β-catenin associates with TCF4 and serves as a transcriptional activator, inducing expression of EMT related transcription factors including SNAI1, ZEB1, and Twist1 [35]. Here we observed that fact that TRIM22 activates Wnt signaling and nuclear β-catenin. Blockage of Wnt signaling abolished the effect of TRIM22 on EMT markers and Snail protein expression. Together these results demonstrate that TRIM22 induces EMT process in NSCLC cells through activation of PI3K/AKT/GSK3β/β-catenin signaling pathway. TRIM22 was also reported as a E3 ubiquitin ligase [11], which assists or directly catalyzes the transfer of ubiquitin to target protein substrate. Certain E3 ubiquitin ligase has been reported to activate AKT signaling through degradation of PTEN [36]. E3 ubiquitin ligase also has been shown to activate or inhibit WNT signaling pathway depending on its target proteins [37–39]. Thus we postulate that TRIM22 may target potential inhibitor of AKT/WNT pathway to exert its biological function. The exact molecular mechanism need further exploration.

In conclusion, this study delineates the clinical significance and biological function of TRIM22 in lung cancer progression. TRIM22 could serve as a valuable prognostic biomarker. TRIM22 promotes NSCLC cell proliferation and invasion through PI3K/AKT/GSK3β/β-catenin mediated EMT process. TRIM22 might be a potential target for the therapeutic strategy against EMT in NSCLC.

## MATERIALS AND METHODS

### Patients and lung cancer samples

The present study was approved by the ethical committee of First affiliated hospital of China Medical University. 126 cases of lung cancer tissue slide were obtained from the first affiliated hospital of china medical university since 2008 to 2012. All procedures performed in studies involving human participants were in accordance with the ethical standards of the institutional and/or national research committee and with the Helsinki declaration and its later amendments or comparable ethical standards. Informed consent was obtained from all individual participants included in the study. All patients underwent curative surgical resection without prior chemotherapy or radiation therapy. For detecting both the messenger RNA (mRNA) and protein expression level of TRIM22, 20 lung cancer and corresponding normal tissues were collected and stored at -80 °C for later RNA or protein extraction.

### Immunohistochemistry

The FFPE tissue sections were treated with xylene, graded alcohol and then antigen retrieval was performed in 0.01 M citrate buffer. hydrogen peroxide was used for blockage. Tissue sections were treated with goat serum for 20 minutes. Then the slides was incubated TRIM22 antibody (1:300, Proteintech, USA) overnight at 4°C. Immunostaining of TRIM22 was carried out using EliVision Super Kit from Maixin (Fuzhou, China). All tumor slides were examined randomly by two independent pathologists. TRIM22 staining was located in both cytoplasmic and nuclear compartment of tumor cells. Nuclear staining was considered as positive staining. Immunostaining of TRIM22 was scored following a semi-quantitative scale by evaluating in representative tumor areas, the intensity and percentage of cells. Five views was randomly selected for each slide and at least 50 tumor cells were evaluated per view. The intensity of TRIM22 staining score was indicated as 0 (no staining), 1 (weak staining), 2 (strong staining). Staining percentage was scored as 0 (0%), 1(1–25%), 2 (26–50%), 3 (51–75%) and 4 (76–100%). Each score was multiplied to a final score of 0 to 8. TRIM22 status was regarded as low TRIM22 expression (score <4) or high TRIM22 expression/overexpression (score≥4).

### Cell culture and transfection

NSCLC cell lines H460, A549, H358, LK2, H1299, H3255 and normal bronchial epithelial cell line NHBE were obtained from ATCC. The cell lines were cultured with RPMI-1640 (Gibco, USA) containing 10 percent bovine serum at 37°C with 5%CO2.

pCMV6-empty vector and pCMV6-TRIM22 plasmid were obtained from Origene company (Origene, Rockville, MD, USA). Plasmid transfection was performed using Attractene (Qiagen, Hilden, Germany) according to manufacturer’s instructions. TRIM22 siRNA, β-catenin siRNA and control siRNA were obtained from Dharmacon (GE healthcare, USA). siRNA transfection was performed using Dharmafect1 reagent (GE healthcare, USA).

### Quantitative real-time PCR

Real-time RT-PCR was carried out using the SYBR Green master mix from ABI (ABI, USA) with a 7500 Real-Time PCR System (ABI, USA). A dissociation step was performed to generate melting curves to confirm the specificity of the amplification. Expression levels of the analyzed genes were normalized to the expression of β-actin. The fold change of gene expression was calculated by the 2^-ΔΔCt^ method. The sequences of the primer pairs are as follows: TRIM22 forward, 5′-GCACGCTCATCTCAGATCTCC-3′; TRIM22 reverse, 5′-TTTTGGCTTCTTCAATGTCCAG-3′. β-actin forward, 5′-CCTCACCCTGAAGTACCCCAT-3′, β-actin reverse, 5′-GCCAGATTTTCTCCATGTCGTC-3′.

### Western blot

Total proteins from cell lines or fresh lung cancer/normal tissues were extracted in lysis buffer and quantified using the Bradford method. 30 μg protein was separated by SDS-PAGE. Samples were transferred to PVDF membranes (Millipore, Billerica, MA, USA) and incubated overnight at 4°C with primary antibodies including TRIM22 (1:800, Proteintech, USA), cyclin D1 (#2978), cyclin E (#4129), p27 (#3686), E-cadherin (#3195), N-cadherin (#13116), Vimentin (#5741), p-GSK3β(#5558), β-catenin (#8480), p-AKT (#4060), AKT (#4691), Snail (#3879), Slug (#9585), Twist (#46702) (1:1000, Cell Signaling Technology, USA) and GAPDH (1:2000, Santa Cruz, USA). After incubation with HRP-coupled anti-mouse or rabbit IgG antibody at 37°C for 2 hours. Target proteins on PVDF membrane were visualized using ECL kit (Pierce) and obtained using DNR Imaging System (DNR, Israel).

### Colony formation and MTT assays

For colony formation assay, cells were transfected for 48 hours and plated into three 6-cm cell culture dishes (1000 cells). Cells were incubated for 2 weeks in medium. Plates were washed with PBS and stained with Giemsa. The number of colonies with more than 50 cells was counted. The colonies were manually counted using a microscope.

For MTT assay, 24 hours after transfection, cells were plated in 96-well plates in at a concentration approximately 2000 cells per well and cultured for 5 days. For quantitation of cell proliferation rate, 20 μl of 5 mg/ml MTT (thiazolyl blue) solution was added to each well and incubated for 4 hours at 37°C. The medium was removed from each well and 150 μl of DMSO was added to the well. The plate was measured at 490 nm.

### Matrigel invasion assay

Matrigel invasion assay was carried out using a 24-well Transwell chamber from Costar (Corning, USA) coated with 20 μl Matrigel with a dilution rate of 1:6 (BD Bioscience, USA). 48 hours after the transfection, cells were trypsinized and transferred to the upper chamber with our serum and incubated for 18 hours. Lower chamber was added with medium supplemented with 10% serum. Non-invaded cells were wiped out and cells invaded through the filter were fixed with 4% paraformaldehyde and stained with hematoxylin.

### Cell cycle analysis

Cells were seeded into 6 cm tissue culture dishes. Forty eight hours after transfection, cells were harvested, fixed in 1% paraformaldehyde, washed with PBS and stained with 5 mg/ml propidium iodide in PBS supplemented with RNase A (Roche, Indianapolis, IN) for 30 minutes at room temperature. Cells in each individual phase of the cell cycle were determined based on their DNA ploidy profile using ACEA Flow Cytometer.

### TOP-Flash Luciferase Reporter assay

TOP-Flash reporter and Renilla luciferase reporter plasmid were transfected into cells with Attractene reagent. About 30 hours after plasmid transfection, dual luciferase reporter kit (Promega, USA) was used to detect luciferase activity.

### Statistical analysis

SPSS version 16 for Windows was used for all statistical analyses. A χ2 test was used to examine possible correlations between TRIM22 expression and clinicopathologic factors. The Kaplan-Meier method was used to estimate the probability of patient survival, and differences in the survival of subgroups of patients were compared by using Mantel’s log-rank test. The Cox regression model was used for multivariate analysis. Student’s t-test was used to compare other data . p value <0.05 was regarded as statistically significant.

## SUPPLEMENTARY MATERIALS FIGURE AND TABLE



## Abbreviations

NSCLC: non-small cell lung cancer
EMT: epithelial-mesenchymal transition
EV: empty vector
