# Supplementary material for: TRIM22 confers poor prognosis and promotes epithelial-mesenchymal transition through regulation of AKT/GSK3β/β-catenin signaling in non-small cell lung cancer
Source: Oncotarget. 2017 Jul 1;8(37):62069–80. doi: 10.18632/oncotarget.18911 (PMC5617487; doi:10.18632/oncotarget.18911)
Supplement: Supplementary file 1 [file oncotarget-08-62069-s001.pdf]

TRIM22 confers poor prognosis and promotes epithelial-mesenchymal transition through regulation of AKT/GSK3β/β-catenin signaling in non-small cell lung cancer

SUPPLEMENTARY MATERIALS

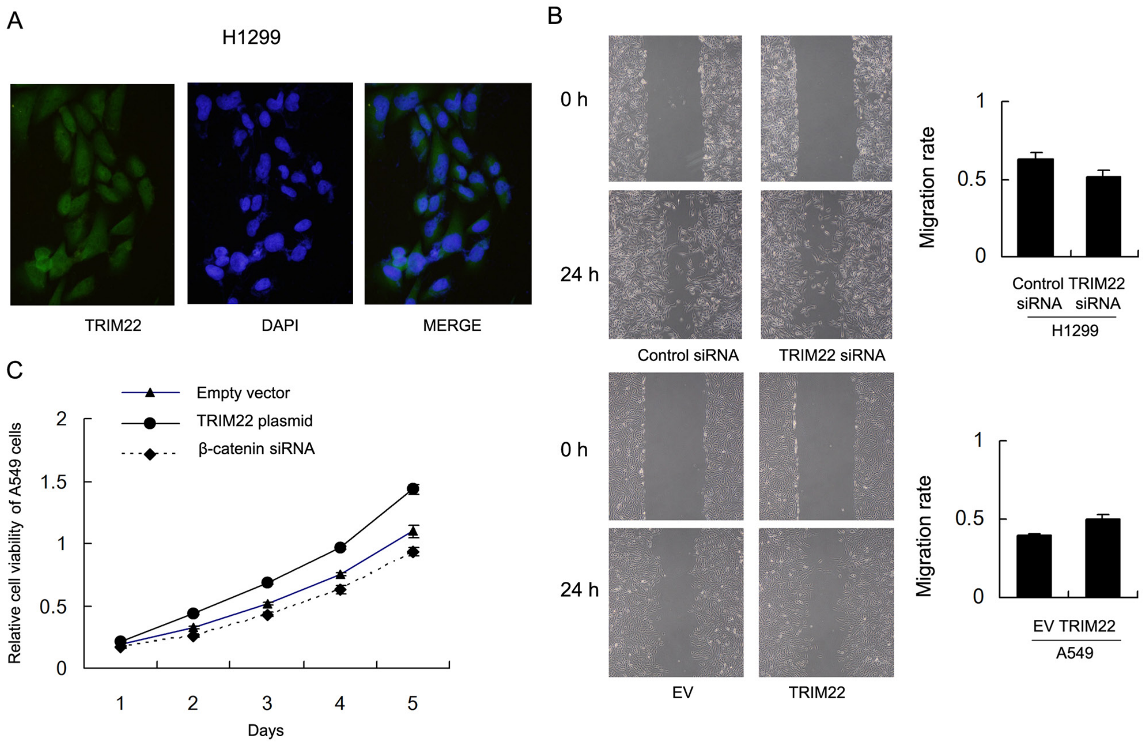

Supplementary Figure 1: The role of TRIM22 on migration and the effect of beta-catenin siRNA on cell growth. (A) Immunocytochemistry of TRIM22 showed both nuclear and cytoplasmic staining in H1299 cell line. **(B)** TRIM22 promoted A549 cell migration while TRIM22 siRNA downregulated migration in H1299 cells. **(C)** TRIM22 transfection upregulated cell growth rate. Beta-catenin siRNA downregulated cell growth rate induced by TRIM22.

Supplementary Table 1: Expression of cytoplasmic TRIM22 in NSCLC

Characteristics	Number of patients	TRIM22 low expression	TRIM22 high expression	*P*
Age				
<60	54	26	28	0.8370
≥60	72	36	46	
Gender				
Male	85	42	43	0.9471
Female	41	20	21	
Differentiation				
Poor	16	9	7	0.4502
Moderate	84	38	46	
Well	26	15	11	
Histology				
Adenocarcinoma	70	31	39	0.2167
Squamous cell carcinoma	56	31	25	
TNM stage				
I	50	32	18	0.0042
П	35	18	17	
ш	41	12	29	
T stage				
T1	31	17	14	0.4700
T2-T4	95	45	50	
Nodal metastasis				
Negative	62	38	24	0.0076
Positive	64	24	40	
